# Raman Spectroscopy of Glass Beads in Ammonium Nitrate Solution and Compensation of Signal Losses

**DOI:** 10.3390/s24020314

**Published:** 2024-01-05

**Authors:** Erik Spoor, Matthias Rädle, Jens-Uwe Repke

**Affiliations:** 1 Center for Mass Spectrometry and Optical Spectroscopy (CeMOS), Mannheim University of Applied Sciences, Paul-Wittsack-Str. 10, 68163 Mannheim, Germany; m.raedle@hs-mannheim.de; 2 Process Dynamics and Operations Group, Technische Universität Berlin, Straße des 17. Juni 135, 10623 Berlin, Germany; jens-uwe.repke@tu-berlin.de

**Keywords:** disperse phase, continuous phase, optical spectroscopy, particle measurement, process control, raman-spectroscopy, process engineering, UV/VIS-spectroscopy, suspension measurement

## Abstract

In the present study, the influence of disperse systems on Raman scattering was investigated. How an increasing particle concentration weakens the quantitative signal of the Raman spectrum is shown. Furthermore, the change in the position of the optimal measurement point in the fluid was considered in detail. Additional transmission measurements can be used to derive a simple and robust correction method that allows the actual concentration of the continuous phase to be determined with a standard deviation of 2.6%.

## 1. Introduction

Process monitoring and analysis is an area that is constantly evolving with increasing digitalization, especially in the chemical, pharmaceutical, and petrochemical industries [1]. Process monitoring methods are becoming increasingly sophisticated and enable a combination of quality-controlled production and increased automation within the framework of process analytics (PAT) [2,3].

Among the process analytical principles and methods, spectroscopic process monitoring is particularly noteworthy. It offers the possibility of measuring inline quality variables for molecular composition directly online and not leaving the control exclusively to process variables such as pressure and temperature [4]. Spectroscopic process measurement technology is divided into UV/VIS spectroscopy, near-infrared, mid-infrared, fluorescence, and Raman spectroscopy. In the field of VIS spectroscopies, applications are used primarily for the differentiation of colors, for example in dye production using ATR spectroscopy. UV or NIR spectroscopy is commonly used for structural composition analysis [4,5,6,7].

Trace substances can be well detected in the ultraviolet radiation range. When a reactant or product shifts its absorption bands to the ultraviolet range, it is possible to monitor the process states. However, the possibilities of UV spectroscopy are limited here because not every molecule has characteristic bands in this wavelength range [8].

Near-infrared spectroscopy was established about 40 years ago and has been widely used since then, but it has reached its limits in terms of the interpretability of multicomponent mixtures and the needed calibration effort [1,9]. The chemometric models used in the analysis of harmonics of molecules applied in near-infrared spectroscopy require extensive training of the measurement equipment or software, which involves significant effort. The change of the matrix, e.g., with change of the supplier for reactants, leads to changed reactant purity and deviating impurities, which result in misinterpretations and thus the requirement of recalibration arises [6,10,11,12,13,14,15]. 

Raman spectroscopy is another measurement technique for process analysis that is slowly gaining acceptance, although considerable research is still required and there is a need for further development. The Raman effect was first discovered in 1928 and has been increasingly used since 1961 after the development of lasers [16]. The considerable development effort in the field of detectors in recent years led to extremely increased detection sensitivity, which is necessary for the weak Raman effect (contribution to the signal about 10^−8^) [6]. The Raman spectrum often shows more discrete bands similar to the mid-infrared spectrum [4,6]. Further advantages are the non-destructive and non-contact measurement process, as well as the high sensitivity and the molecule-specific spectra [17]. The disadvantage of the method lies in the lasers to be used, whose necessary power can range up to 500 mW and collide with explosion protection requirements (DIN EN 60079-28) [18]. Where possible, however, significantly lower laser powers are used today, in the range of 10 mW or even down to 0.1 mW [19,20]. High laser powers can also cause decomposition of the target in light-sensitive samples [17]. In addition, considerable safety precautions have to be taken according to DIN EN 60825-1 for the protection of people and eyesight. In the field of Raman spectroscopy, there are already well-established areas of application, such as the monitoring of organic liquids and areas that unfortunately still elude quantitative analysis at present.

In this study, processes with disperse systems are discussed. Such processes include emulsions, i.e., droplets in a fluid continuous matrix, suspensions, i.e., particles in a continuous fluid matrix, aerosols, bubble columns, bubble-containing substance systems, and mixtures thereof, such as Suspo-emulsions, which are used in plant protection [21,22,23,24]. The model system of the suspension investigated in this study consists of an ammonium nitrate solution as the continuous phase and glass beads with an average diameter of 2 μm as the disperse phase. The challenge here lies in the fact that the backscattered Raman light’s intensity collapses when a second phase occurs with the result that interpretation is no longer possible. However, since many procedural processes are multiphase rather than single-phase, this paper focuses on first steps in the quantitative analysis of Raman-measured dispersed systems and the development of a solution for signal correction.

An example of the application of Raman spectroscopy for the investigation of multiphase systems would be polymerization. Polymers are used in the automotive industry, construction, electronics, agriculture, or household products, for example, resulting in a wide range of applications [25]. To monitor the reaction process and the quality of the reaction, it is necessary to measure the product. This can be done using offline measurement methods, such as headspace gas chromatography, but such methods only provide results with a time delay and are only momentary impressions of the reaction [26]. A more practical solution is provided by online measurement methods, including Raman spectroscopy. The Raman probe can follow the reaction process non-invasively and, as no sample has to be taken (as with offline methods), also provides a non-destructive alternative [27,28]. The problem with spectroscopy, as described above, is that, in multiphase systems, the detected signal can drop significantly, and new dependencies arise due to the interfaces between the individual phases. Therefore, it is usually necessary to create complex regression models that take all variables, such as particle size, into account. These methods offer the advantage of good quantitative results but require complex and time-consuming calibrations for the reaction in question [29,30]. This study, however, is intended to offer a simpler solution in which the Raman measurement is combined with a UV/VIS measurement. The aim was to create a model with a straightforward calibration, which works without the necessity of knowing other parameters such as particle size.

Other general problems are addressed, for example, in the work of Hufnagel et al. or Kollhoff et al., where solution approaches in the form of refractive index matching are used, minimizing the influence of the boundary layers [31,32]. The drawback is that refractive index matching requires changing the composition of at least one phase. Other works, such as those by Meyer et al., Schalk et al., and van den Brink et al., deal with targeted calibration models based on peak ratios being constant in the spectra [33,34,35]. For more complex systems, where the peak ratios can also change or where it is not possible to detect a reference peak, a general solution has not yet been found. The general influences of boundary layers and refractive index differences between the phases on incident light have already been well investigated [36]. The aim of our work was to find a more general approach for the correction of signal losses in disperse systems that is independent of the individual systems.

In this study, a suspension that provides the advantage of a constant particle size distribution and thus easily reproducible measurement results was investigated. The chosen approach uses a fluid system without its own Raman bands. The focus of the work is on the measurement of the continuous fluid phase, the recognition of the correlations and changes in the case of an occurrence of dispersed phases, and the attempt to work out a multiparametric approach for the correction. Furthermore, guidance is provided on how to reduce the influence of the dispersed phase on the measurement of the continuous phase as much as possible in addition to the correction. Several series of experiments were performed to determine how the signal changes with variation in the measuring point and the concentration of the glass beads used, and a simple correction for partial areas was finally determined. The calculation of the measurement signal with the correction function compensates for the signal losses and thus provides an output of the expected nominal curve.

## 2. Materials and Methods

**Figure 1:**
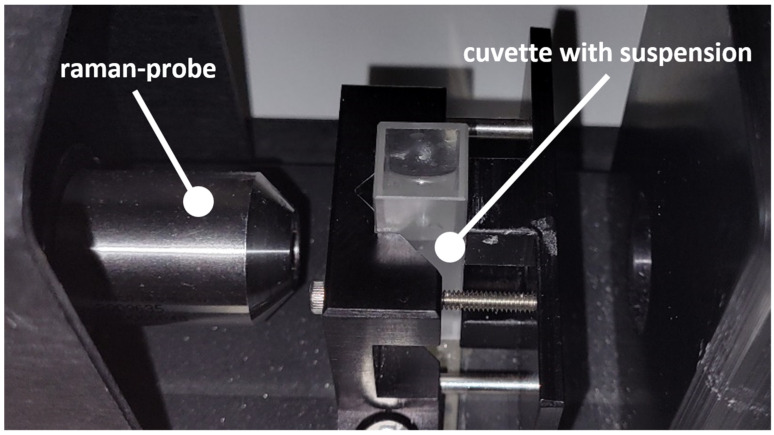
Measurement setup of the probe fixed vertically in front of the cuvette.

A Raman-RXN1 spectrometer and a Raman-Rxn-10 probe (NCO-0.5-VIS) from Kaiser Optical Systems (Ann Arbor, MI, USA) Inc. were used for the measurements. A laser with 150 mW power and an excitation wavelength of 532 nm was installed in the spectrometer. The probe, which measures in a cuvette with a focal length of 12.5 mm, an F/number of 2.0, and a spot size of approximately 200 µm can be connected via a fiber coupling. A measurement chamber from Kaiser Optical Systems was used to set a reproducible coupling of the laser (Figure 1). The probe was mounted in the chamber in a position so that it could only be moved on one axis and could thereby move closer to or further away from the cuvette, which was fixed in position. The cuvettes used were macro cuvettes from Hellma (Müllheim, Germany). The cuvettes were made of quartz glass and were designed for a wavelength range of 200–2500 nm. The layer thickness was 10 mm, with a wall thickness of 1.25 mm, which the laser had to pass through. Once the probe was inserted into the measurement box up to its mark, the focal point was in the center of the cuvette. Excitation of the sample occurred at the focal point, and the backscattered light was collimated by the probe and detected by a second fiber coupling in the spectrometer. The detection range covered the Raman spectrum from 0 to 4400 cm^−1^, with the excitation wavelength filtered out to about 40 cm^−1^.

The fluid system used was an ammonium nitrate (98 wt %, Sigma Aldrich (St. Louis, MO, USA)) solution [37] with Omicron NP3 P0 glass beads (SiO_2_) from Sovitec (Fleurus, Belgium). For the glass beads used, a size distribution and the diameter were already determined in the work of Schmitt et al. using a HELOS particle size analyzer from Sympatec (Clausthal-Zellerfeld, Germany) [38]. The Sauter diameter was 2.093 µm. The glass beads represented the disperse phase, the proportion of which in the total system ranged from 0.00 to 3.14 wt %. The continuous phase consisted of ammonium nitrate, 20.00 wt % of which was dissolved in deionized water. The ratio between ammonium nitrate and deionized water, or rather the composition of the continuous phase, was constant for all samples. The proportions of the mixtures of the respective samples are listed in Table 1.

The refractive index of a 20.00 wt % ammonium nitrate solution, which can be measured with an ORF-E digital refractometer from Kern & Sohn GmbH (Balingen, Germany), was 1.359, and the refractive index of the glass beads was 1.457. The difference leading to light refraction therefore was 0.098. This is comparable, for example, to an emulsion of dodecene and water (difference of 0.096). One emulsion system, namely, silicone oil-water, has a lower difference of 0.069, and another, namely, toluene-water, has a higher difference of 0.129. Thus, the light refraction in the suspension studied in this paper is of a comparable scale to that in other disperse systems.

**Figure 2:**
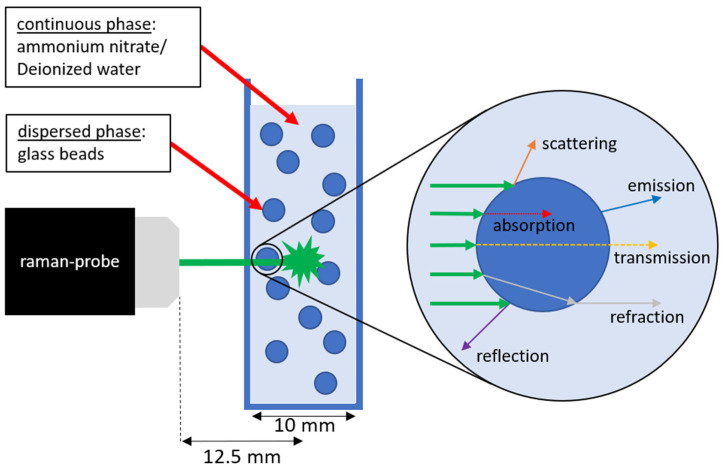
Scheme of probe position and light scattering by glass beads.

As the concentration or number of glass beads increases, so does the number of optical boundary layers with scattering effects through which the laser must pass. For each boundary layer, the interactions between laser light and glass beads shown in Figure 2 occur, in which the light is deflected from its original direction and/or attenuated [36]. To investigate these light losses more closely, measurements were first carried out with varying positions of the focal point to find an optimal measurement position, and correction functions for the signal losses in the selected position were then created.

**Figure 3:**
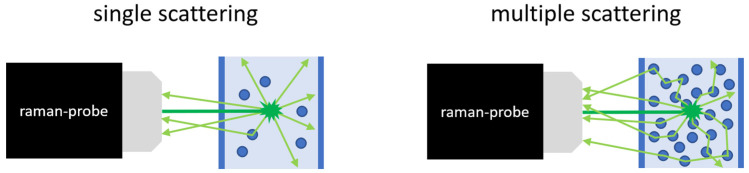
Single and multiple scattering of the Raman signal in emulsions and suspensions.

In disperse systems, multiple scattering occurs due to the high number of particles (Figure 3). In this process, light that has already been deflected is deflected again from its path. There is also the possibility that light can be scattered back to the detector compared to single scattering. As a result, as the particle concentration increases, the light losses increase more slowly and the sum of the losses enters saturation [36,39].

**Figure 4:**
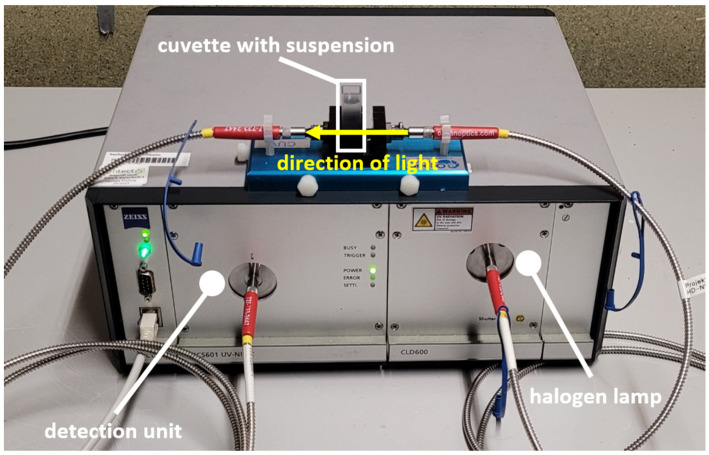
Setup for transmission measurement in a cuvette with a UV spectrometer.

In addition to Raman spectroscopy, transmission arrangement measurements were carried out at each concentration, and a Zeiss (Oberkochen, Germany) spectrometer was used, as seen in Figure 4. The modules installed were an MCS 601 UV-NIR spectrometer cassette and a CLD 600 lamp cassette. The lamp cassette was equipped with a halogen lamp that continuously emits a spectral range of 210–600 nm with a radiation power of 2.5 mW. The spectrometer cassette enabled the detection of the wavelength range from 190 to 1015 nm with a resolution of 0.5 nm. The lamp was coupled via an optical fiber in front of the same cuvettes as for the Raman measurement. The light was emitted freely into the cuvette and detected on the opposite side with another optical fiber connected to the detection unit of the spectrometer.

## 3. Results

**Figure 5:**
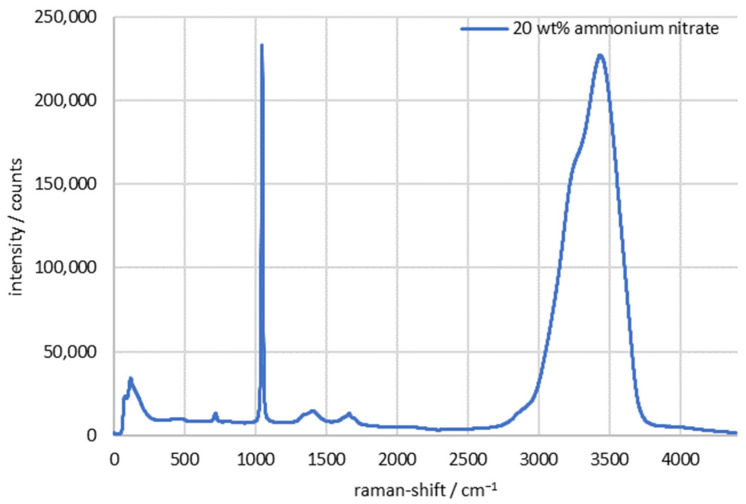
Raman spectrum of 20 wt % ammonium nitrate in deionized water/integration time: 2.5 s/accumulation: 5.

Figure 5 shows a typical Raman spectrum of a 20 wt % ammonium nitrate solution. The characteristic peak of ammonium nitrate, i.e., the NO_3_^−^ vibrational band, is found at 1047 cm^−1^. The measurement result also matches spectra that can be found in the literature [31,40]. The peak heights ($I_{AN}$) of ammonium nitrate read from this spectrum were calculated according to Formula 1 by subtracting the average of the left ($I_{987{cm}^{-1}}$) and right ($I_{1107{cm}^{-1}}$) peak bases from the highest point ($I_{1047{cm}^{-1}}$).

(1) $$I_{AN}=I_{1047{cm}^{-1}}-\frac{I_{1107{cm}^{-1}}+I_{987{cm}^{-1}}}{2}$$

No separate Raman measurement was performed for the glass beads, but it can be demonstrated from literature research that there is no significant cross influence from other bands of SiO_2_ in the region of the ammonium nitrate peak [41,42].

In the following evaluations, only the change in the peak height as a function of particle concentration is shown in each case.

**Figure 6:**
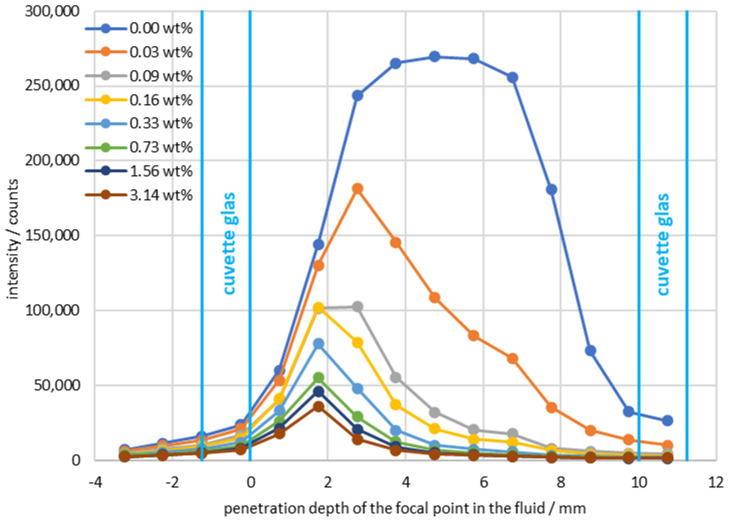
Intensity of the characteristic ammonium nitrate peak plotted against the position of the focal point/ammonium nitrate concentration: 20 wt %/integration time: 2.5 s/accumulations: 5.

Figure 6 shows curves of measured ammonium nitrate count rates with a changing focus in the suspension. The *x*-axis is scaled so that the inside of the glass corresponds to a focal point intrusion depth of 0 mm. To illustrate the focus position, the cuvette walls are marked as vertical lines in the graph. The top curve shows the course of a pure ammonium nitrate solution, while in the lower curves the particle concentration was systematically increased. An increasing particle concentration led to a drop in the signal, independent of the focal point. Moreover, if the focal point is deeper in the suspension, the signal is further weakened. In the case of suspensions above 0.03 wt %, there was one optimal measurement position per curve at a penetration depth of 1.75 mm. For lower concentrations towards the pure ammonium nitrate solution, the maximum of the signal shifted to the center of the cuvette.

For a pure ammonium nitrate solution, there was even a plateau with a width of approximately 3 mm. This means that the optimal measuring position for measurement with particles was different from that for measurement without particles. The drop in the measured count rate related to the ammonium nitrate concentration when moving the focus did not show a jump function, but a gradual transition. The transition can be explained by the focus position of the probe used, which had a certain depth of field, here approximately 3 mm. When the depth of the penetration of the focus into the solution was reduced, partly the solution was measured and partly the light was found inside the front glass wall. The closer the focal point was to the front wall, the less the count rates changed with the increasing particle concentration in the solution. This can easily be explained by the beam path that required less penetration of the suspension for these measurements than for measurements at a depth of 5 mm, for example.

**Figure 7:**
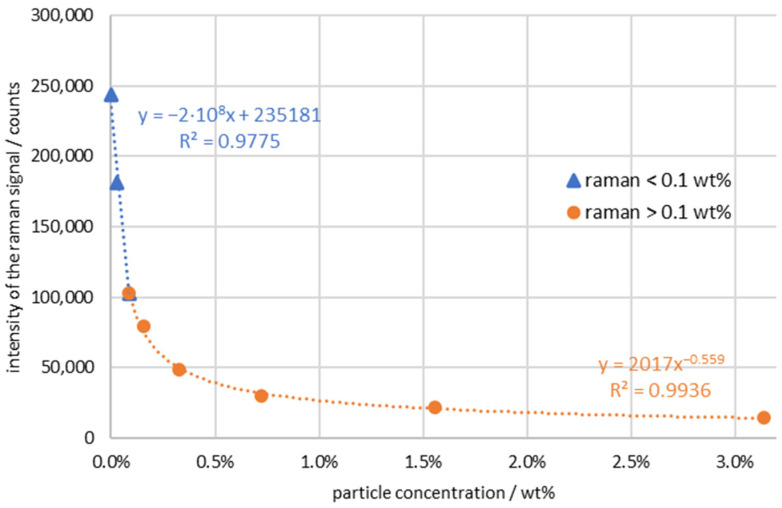
Raman intensity of ammonium nitrate plotted against particle concentration/ammonium nitrate concentration: 20 wt %/integration time: 2.5 s/accumulations: 5.

For further evaluation, a measurement series with a penetration depth of 2.75 mm was chosen, which represents a compromise between the previously determined optimal position for concentrations below 0.03 wt % and the higher concentrations. A closer look at the results of the Raman measurement in Figure 7 shows that two concentration ranges can be distinguished. For particle concentrations below 0.1 wt %, the curves of the measured intensity versus the particle concentration can be interpolated approximately linearly, as shown in Figure 7 below. The picture changes when the particle concentration is higher than 0.1 wt %. In this case, the graph can rather be represented by an exponentially decreasing curve. The observation can be explained in detail by looking at the scattering and absorption processes. The light was emitted from the probe into the sample, penetrated the suspension, and was attenuated by scattering and reflection at the surfaces of the particles. As a result, the excitation light was deflected from the primary direction on its way to the focal point. In the focus point, a scattering process took place, and the light therein was scattered by the molecule. In a Raman measurement, the light excites the molecule into a virtual state. After a very short time, such as 10–16 s, the excited molecule falls back to the ground state and emits light isotopes in all directions with a different wavelength. This light is the result of the curves shown. The light passes through the suspension on its way back and arrives at the detector. There are two types of processes: One is the penetration of a suspension, a process that is like a transmission measurement, such as in the near infrared. As expected, the Beer–Lambert law is partially applicable here. The scattering process itself is comparable to elastic scattering (see Guffart et al. [43]) and shows a linear behavior on the intensity scale with the change in concentration of ammonium nitrate and the integration time. In addition, with increasing particle concentration, further scattering occurs, which causes the measured signals to deviate from the linearity of the Beer–Lambert law, which results in an exponential decrease. The total signal is therefore the sum of an exponential curve with a negative exponent and a linear curve. In the small concentration ranges, the exponential curve attenuates the light only insignificantly. The dynamics of the linear scattering outweigh the measurement curves. When the particle concentration is increased, the exponential curve becomes increasingly apparent. The two curves are shown in Figure 7.

**Figure 8:**
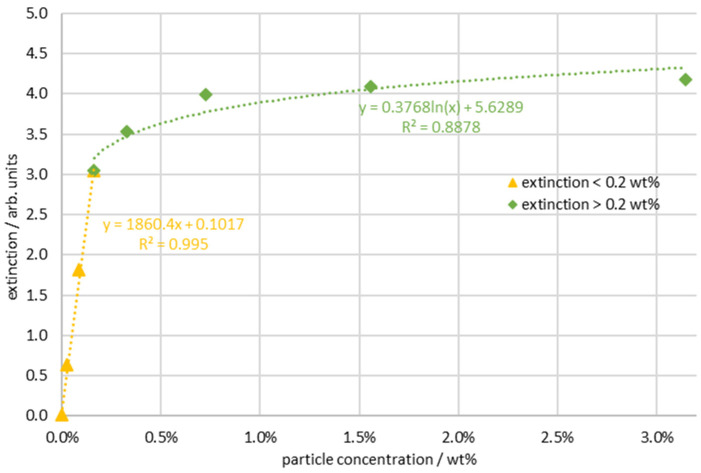
Extinction measurement of the suspension samples with transmission arrangement with a 10 mm layer thickness.

In order to calibrate the scattering processes principally occurring in particulate systems and compare them with other phenomena, transmission measurements were carried out with the same suspensions and in the same cuvettes (layer thickness: 10 mm), and the attenuation in the wavelength range of 561–565 nm was evaluated classically and presented as extinction. In Figure 8, the blue curve initially shows a linear increase in extinction $E_{\lambda }$, as expected from the Beer–Lambert law [4] (Formula (2)). It is calculated as the logarithm of the ratio between the light intensity that enters the sample ($I_{0}$) and the light that exits the sample ($I_{1}$). Alternatively, the extinction can be described as the product of the molar absorption coefficient ($\varepsilon_{\lambda }$), the concentration of the sample (c), and the optical path length (d).

(2) $$E_{\lambda }=\log_{10}\left(\frac{I_{0}}{I_{1}}\right)=\varepsilon_{\lambda }\cdot c\cdot d$$

The calculation of the extinction via the Beer–Lambert law results in a linear dependence on the concentration or the layer thickness. The curve deviates from the linearity of the Beer–Lambert law when the particle concentration increases above approximately 0.2 wt % (the green curve in Figure 8). Above this concentration, multiple scattering processes predominate. This means that the irradiated light, which is scattered out of the primary beam, can also be scattered back into the primary direction by another particle involved and another scattering process. As a result, there is a massive deviation from the linear course on the extinction scale. The concentration of the breakpoint corresponds to the concentration that separates the two areas in the Raman measurement. It is therefore obvious that the Raman measurement in the range of the smaller concentrations below a maximum of 0.1 wt % can be explained via Beer–Lambert attenuation factors and single scattering. Therefore, the correction function of the transmission measurement offers the possibility of correcting the Raman measurement in this concentration range.

The correction function can be created by plotting the change in the Raman peak against the extinction per millimeter of layer thickness. The calculation is done according to Formula (3) by putting the intensity of the signal I in relation to the signal of the sample without particles $I_{0}$.

(3) $$∆I=\frac{I_{0}}{I}$$

**Figure 9:**
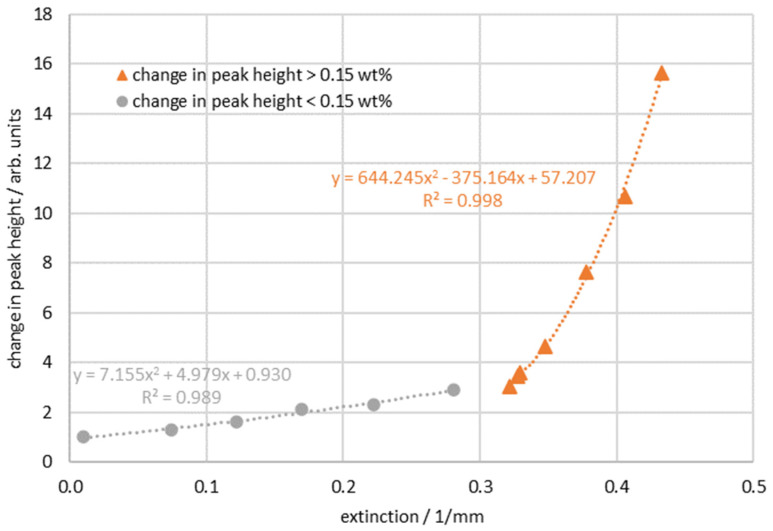
Change in the Raman peak plotted against the extinction.

The resulting ∆I is plotted against the extinction in Figure 9. Analogous to Figure 7 and Figure 8, two areas emerge that can be described by a quadratic regression. The limit is approximately 0.15 wt %, which corresponds to the mean value of the limits of the Raman measurement (0.1 wt %) and the extinction measurement (0.2 wt %). This results in correction Formulas (4) and (5) via which a correction factor (${∆I}_{<0.15wt\%}\;$ and ${∆I}_{>0.15wt\%}$) for the Raman signal can be calculated using the simultaneously measured extinction E:

(4) $${∆I}_{<0.15wt\%}=7155\cdot E^{2}+4979\cdot E+0.930$$

(5) $${∆I}_{>0.15wt\%}=644,245\cdot E^{2}-375,164\cdot E+57,207$$

The correction factor ∆I for the measured concentration can then be multiplied by the associated Raman measurement $I_{AN}$ to correct the signal ($I_{AN-corr}$) (Formula (6)):

(6) $$I_{AN-corr}=I_{AN}\cdot ∆I$$

**Figure 10:**
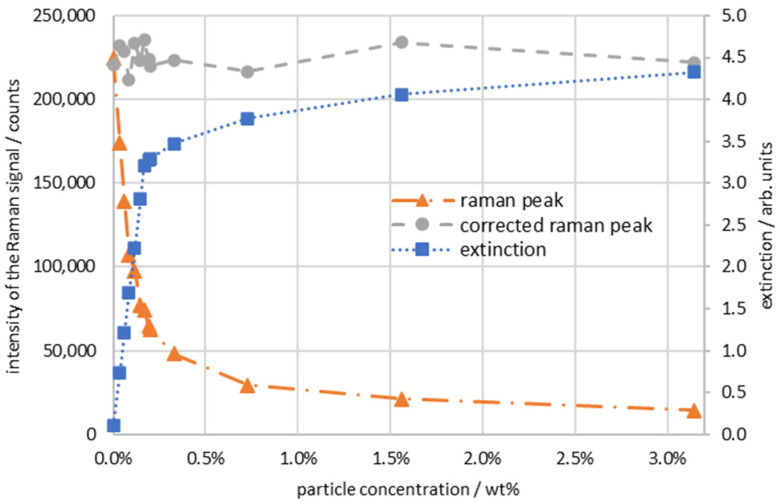
Intensity, corrected intensity, and extinction of the Raman signal of ammonium nitrate plotted versus particle concentration.

This correction is made in Figure 10, and the result is shown by the grey curve. In the measured area, the original Raman count rate is corrected to give the expected ammonium nitrate concentration of the constant 20 wt %, with a mean standard deviation of 2.6% as the result. For the correction function up to 0.15 wt %, the mean standard deviation is 2.9%; for the higher concentrations, it is 2.4%.

Based on the measurements made so far, we can draw two conclusions that will have to be further investigated in the future. Firstly, it is favorable to carry out the Raman measurement in the otherwise suboptimal area on the front glass wall. Secondly, it is suitable to find a correction value by measuring a transmission signal in the elastic backscattering. With this correction value, it is possible to correct the Raman measurement scattered by the disperse phase. These conclusions are applicable for the time being under the condition of a constant particle size distribution and in the considered concentration range up to about 3.14 wt %. To further generalize the results, additional studies must be done, which take into account a variation of all parameters.

## 4. Discussion and Conclusions

The aim of this study was to compensate for the signal losses that occur during Raman spectroscopic measurements of a suspension of glass beads in an ammonium nitrate solution. To achieve this, reference measurements were performed with a halogen lamp in transmission. By combining both signals, the measured data of the Raman measurement can be adjusted to such an extent that the standard deviation results in a value of 2.6%. For a future measurement in a process, it could be realized by a simple transmission probe and halogen lamp, which could be installed in addition to the Raman probe. The linearity of the Raman and transmission measurements plotted against the lower concentration of glass beads can be explained by the Beer–Lambert law, which describes a linear relationship between the extinction of light and the concentration of the sample. At higher concentrations (for the particle size considered in this work: >0.1 wt % for Raman and >0.2 wt % for transmission), effects of multiple scattering were added, and such effects predominate and therefore cause the measurement curves to deviate from the linear progression of the Beer–Lambert law. The multiple scattering leads to the effect that even light that has already been deflected can be scattered back to the detection and thus the course of the curve flattens out. Therefore, a correction via a single correction function is not possible and two ranges must be defined. The combination of Raman and extinction measurement results in a transition range at a particle concentration of 0.15 wt %.

All present measurements were made under the condition of a constant concentration (20 wt %) of the continuous phase and with a constant Sauter diameter of the particles (2.093 µm). The current calibration was also carried out at one specific penetration depth of the focal point. In future work, series of measurements should be performed where these parameters are also varied to obtain a more detailed data matrix on the influences affecting the measurement signal. A general calibration should then be developed, which allows for a correction of the Raman signal using as little additional information as possible and thus minimizes the cross-influences of dispersions.

## Figures and Tables

**Table 1:** Concentrations of the fluid system components in weight percent.

Continuous Phase		Dispersed Phase
Ammonium Nitrate/wt %	Deionized Water/wt %	Glass Beads/wt %
20.00	80.00	0.00
19.99	79.97	0.03
19.99	79.95	0.06
19.98	79.93	0.09
19.98	79.91	0.11
19.97	79.88	0.15
19.97	79.87	0.17
19.96	79.85	0.19
19.96	79.84	0.20
19.96	79.71	0.33
19.88	79.39	0.73
19.72	78.72	1.56
19.40	77.46	3.14

## Data Availability

The data presented in this study are available from the corresponding author upon request.

## References

[1] Bakeev K.A. (2010). Process Analytical Technology: Spectroscopic Tools and Implementation Strategies for the Chemical and Pharmaceutical Industries.

[2] Kessler R.W., Kessler W., Zikulnig-Rusch E. (2016). A Critical Summary of Spectroscopic Techniques and their Robustness in Industrial PAT Applications. Chem. Ing. Tech..

[3] Simon L.L., Pataki H., Marosi G., Meemken F., Hungerbühler K., Baiker A., Tummala S., Glennon B., Kuentz M., Steele G. (2015). Assessment of Recent Process Analytical Technology (PAT) Trends: A Multiauthor Review. Org. Process Res. Dev..

[4] Parson W.W.B.C. (2023). Modern Optical Spectroscopy: From fundamentals to Applications in Chemistry, Biochemistry and… Biophysics.

[5] Ritgen U. (2020). Analytische Chemie I.

[6] Günzler H. (2003). IR-Spektroskopie: Eine Einführung.

[7] Vahur S., Knuutinen U., Leito I. (2009). ATR-FT-IR spectroscopy in the region of 500–230 cm^−1^ for identification of inorganic red pigments. Spectrochim. Acta A Mol. Biomol. Spectrosc..

[8] Shinde G., Godage R.K., Jadhav R.S., Manoj B., Aniket B. (2020). A Review on Advances in UV Spectroscopy. Res. J. Sci. Technol..

[9] Blanco M., Villarroya I. (2002). NIR spectroscopy: A rapid-response analytical tool. TrAC Trends Anal. Chem..

[10] Wang H.-P., Chen P., Dai J.-W., Liu D., Li J.-Y., Xu Y.-P., Chu X.-L. (2022). Recent advances of chemometric calibration methods in modern spectroscopy: Algorithms, strategy, and related issues. TrAC Trends Anal. Chem..

[11] Pandiselvam R., Mahanti N.K., Manikantan M.R., Kothakota A., Chakraborty S.K., Ramesh S.V., Beegum P.S. (2022). Rapid detection of adulteration in desiccated coconut powder: Vis-NIR spectroscopy and chemometric approach. Food Control.

[12] Li W., Luo Y., Wang X., Gong X., Huang W., Wang G., Qu H. (2022). Development and Validation of a Near-Infrared Spectroscopy Method for Multicomponent Quantification during the Second Alcohol Precipitation Process of Astragali radix. Separations.

[13] Quintero Balbas D., Lanterna G., Cirrincione C., Fontana R., Striova J. (2022). Non-invasive identification of textile fibres using near-infrared fibre optics reflectance spectroscopy and multivariate classification techniques. Eur. Phys. J. Plus.

[14] Büning-Pfaue H. (2003). Analysis of water in food by near infrared spectroscopy. Food Chem..

[15] Shao X., Bian X., Liu J., Zhang M., Cai W. (2010). Multivariate calibration methods in near infrared spectroscopic analysis. Anal. Methods.

[16] Krishnan R.S., Shankar R.K. (1981). Raman effect: History of the discovery. J. Raman Spectrosc..

[17] Vaskova H. (2011). A powerful tool for material identification: Raman spectroscopy. Int. J. Math. Models Methods Appl. Sci..

[18] Braun F., Schwolow S., Seltenreich J., Kockmann N., Röder T., Gretz N., Rädle M. (2016). Highly Sensitive Raman Spectroscopy with Low Laser Power for Fast In-Line Reaction and Multiphase Flow Monitoring. Anal. Chem..

[19] Ma B., Rodriguez R.D., Ruban A., Pavlov S., Sheremet E. (2019). The correlation between electrical conductivity and second-order Raman modes of laser-reduced graphene oxide. Phys. Chem. Chem. Phys..

[20] Makukha O., Lysenko I., Belarouci A. (2023). Liquid-Modulated Photothermal Phenomena in Porous Silicon Nanostructures Studied by μ-Raman Spectroscopy. Nanomaterials.

[21] Gutiérrez T.J. (2019). . Polymers for Agri-Food Applications.

[22] Ohkouchi T., Tsuji K. (2022). Basic Technology and Recent Trends in Agricultural Formulation and Application Technology. J. Pestic. Sci..

[23] Bibette J., Calderon F.L., Poulin P. (1999). Emulsions: Basic principles. Rep. Prog. Phys..

[24] Schramm L.L. (2014). Emulsions, Foams, Suspensions, and Aerosols: Microscience and Applications.

[25] Boudenne A., Ibos L., Candau Y., Thomas S. (2011). . Handbook of Multiphase Polymer Systems.

[26] Zhong J.-F., Chai X.-S., Fu S.-Y., Qin X.-L. (2012). An improved sample preparation method for monomer conversion measurement using headspace gas chromatography in emulsion polymerization research. J. Appl. Polym. Sci..

[27] Martínez R.I., Ramírez A.O., González R.L., León R.D.d., Cárdenas L.V., Martínez E.T., Elizondo A.D., Vielma B.R. (2017). Polymerization Reactor Monitoring by In-line Raman Spectrometry. J. Mater. Sci. Eng. A.

[28] Frauendorfer E., Hergeth W.-D. (2017). Industrial application of Raman spectroscopy for control and optimization of vinyl acetate resin polymerization. Anal. Bioanal. Chem..

[29] Chen X., Laughlin K., Sparks J.R., Linder L., Farozic V., Masser H., Petr M. (2015). In Situ Monitoring of Emulsion Polymerization by Raman Spectroscopy: A Robust and Versatile Chemometric Analysis Method. Org. Process Res. Dev..

[30] Chang C., Feng L.-F., Gu X.-P., Zhang C.-L., Dai L.-K., Chen X., Hu G.-H. (2022). In Situ Raman Spectroscopy Real-Time Monitoring of a Polyester Polymerization Process for Subsequent Process Optimization and Control. Ind. Eng. Chem. Res..

[31] Hufnagel T., Rädle M., Karbstein H.P. (2022). Influence of Refractive Index Differences on the Signal Strength for Raman-Spectroscopic Measurements of Double Emulsion Droplets. Appl. Sci..

[32] Kollhoff R.T., Kelemen K., Schuchmann H.P. (2015). Local Multiphase Flow Characterization with Micro Particle Image Velocimetry Using Refractive Index Matching. Chem. Eng. Technol..

[33] Schalk R., Braun F., Frank R., Rädle M., Gretz N., Methner F.-J., Beuermann T. (2017). Non-contact Raman spectroscopy for in-line monitoring of glucose and ethanol during yeast fermentations. Bioprocess Biosyst. Eng..

[34] van den Brink M., Pepers M., van Herk A.M. (2002). Raman spectroscopy of polymer latexes. J. Raman Spectrosc..

[35] Meyer K., Ruiken J.-P., Illner M., Paul A., Müller D., Esche E., Wozny G., Maiwald M. (2017). Process spectroscopy in microemulsions—Setup and multi-spectral approach for reaction monitoring of a homogeneous hydroformylation process. Meas. Sci. Technol..

[36] Kortüm G. (2014). Reflectance Spectroscopy: Principles, Methods, Applications.

[37] Carl Roth Safety Data Sheet Ammonium Nitrate. https://www.carlroth.com/medias/SDB-X988-GB-EN.pdf?context=bWFzdGVyfHNlY3VyaXR5RGF0YXNoZWV0c3wyNjIxNTF8YXBwbGljYXRpb24vcGRmfHNlY3VyaXR5RGF0YXNoZWV0cy9oNjUvaDFiLzkwMTUzMDQyMjQ3OTgucGRmfDZjZGU1NDNjNTUyOGRlMDE3OGQzMGIzODE2ZWUwMTM1YTRlZTkxMzIzMDYxMDk5MjIzZjYyYzM3YWI3YjJiMjY.

[38] Schmitt L., Meyer C., Schorz S., Manser S., Scholl S., Rädle A.M. (2022). Use of a Scattered Light Sensor for Monitoring the Dispersed Surface in Crystallization. Chem. Ing. Tech..

[39] Mishra Y.N. (2018). Droplet Size, Concentration, and Temperature Mapping in Sprays Using SLIPI-Based Techniques.

[40] Gillen G., Najarro M., Wight S., Walker M., Verkouteren J., Windsor E., Barr T., Staymates M., Urbas A. (2015). Particle Fabrication Using Inkjet Printing onto Hydrophobic Surfaces for Optimization and Calibration of Trace Contraband Detection Sensors. Sensors.

[41] Henderson G.S., Neuville D.R., Cochain B., Cormier L. (2009). The structure of GeO2–SiO2 glasses and melts: A Raman spectroscopy study. J. Non-Cryst. Solids.

[42] Chen X., Feng W., Zhang G., Gao Y. (2019). Raman Spectra of Quartz and Pb4+-Doped SiO2 Crystals at Different Temperature and Pressure. Crystals.

[43] Guffart J., Bus Y., Nachtmann M., Lettau M., Schorz S., Nieder H., Repke J.-U., Rädle M. (2020). Photometric Inline Monitoring of Pigment Concentration in Highly Filled Lacquers. Chem. Ing. Tech..

